# Risk of relapse: TB treatment outcome associates with differentially culturable *M. tuberculosis* counts in sputum samples

**DOI:** 10.5588/ijtldopen.25.0085

**Published:** 2025-08-13

**Authors:** J. Decker, G.V. Mukamolova, N. Garton, D.J. Grint, M.R. Barer

**Affiliations:** ^1^ Leicester Tuberculosis Research Group, Department of Respiratory Sciences, University of Leicester, Leicester, UK;; ^2^ National Institute for Health and Care Research Leicester Biomedical Research Centre, Leicester, UK;; ^3^ School of Health and Social Care, University of Gloucestershire, UK;; ^4^ London School of Hygiene and Tropical Medicine, Department of Infectious Disease Epidemiology, London, UK; ^5^ Department of Clinical Microbiology, University Hospitals of Leicester.

**Keywords:** tuberculosis, *Mycobacterium tuberculosis*, antibiotic tolerant bacilli, persisters, treatment outcome

Dear Editor,

Prediction of TB treatment outcomes early during therapy is highly desirable both in the management of individuals and in clinical trials. Although disease free status following six months of standard treatment provides an important frame of reference, it is recognised that some patients with drug sensitive TB relapse, with a return to active disease due to their original *Mycobacterium tuberculosis* (Mtb) strain. The frequency of relapse in the face of acceptable treatment adherence is not easily determined, and requires strain genome sequence analysis, but is estimated at 5%.^1^ In recent clinical trials, direct assessment of relapse rates established with 6–12 months follow-up are compared between standard treatment and test regimens to determine non-inferiority. Although the objective of shorter regimens is to achieve equivalent success rates to standard 6-month treatment it is generally recognised that shorter treatment is often associated with higher relapse rates.^2^ The accepted standard of 6–12 months follow-up to detect relapses places significant resource burden on clinical trials to differentiate between relapse and new infection, to assess programme success. Analyses capable of predicting unsatisfactory treatment outcome, including relapse, at or subsequent to treatment initiation, would therefore be extremely beneficial.

Multiple factors have been associated with TB relapse, and the ability of Mtb to form antibiotic tolerant bacilli, referred to in this context as ‘persisters’, is considered a major factor.^3^ Persisters, which survive antimicrobial exposure in vitro, are thought to contribute both to the need for prolonged treatment and subsequent relapse.^4^ We previously suggested that microscopy-based enumeration of lipid bodies containing Mtb cells in sputum (a persister-like property) might associate with unsatisfactory treatment outcomes.^5,6^ We subsequently obtained evidence supporting this in a clinical study.^7^ Although microscopy requires AFB positive sputum, differentially culturable Mtb (DCMtb) associated with a persister phenotype, are readily detected at levels down to a few bacilli per sample.^8^ DCMtb do not form colonies when inoculated onto solid media but, in liquid media supplemented with culture supernatant (CSN) harvested from exponentially growing Mtb cultures, resuscitate and grow to visible densities and can be enumerated by limiting dilution most probable number (MPN) assays. CSN contains the resuscitation promoting factor (Rpf) molecules, principally responsible for resuscitation activity but also other, yet to be defined, activities contribute.^9^ The biological activities of purified Rpfs defy stable storage, thus CSN remains the material used to demonstrate DCMtb. Relative numbers of DCMtb are quantified by the resuscitation index (RI) which reflects the excess of bacilli enabled to grow by CSN over those forming colonies (see Figure). DCMtb clearly associate with TB relapse in mice,^10^ and are shown to be tolerant to certain drugs.^8,11^ Furthermore, DCMtb have been detected in higher proportions in sputum from treated patients,^12–15^ suggesting their potential contribution to unfavourable treatment outcomes, but there is no direct evidence for this. One study described detection of DCMtb after the successful completion of treatment^12^ and a separate project demonstrated that TB sputum from patients with unfavourable outcomes always had DCMtb.^15^ However, low numbers of TB relapses in both studies precluded a robust evaluation of DCMtb abundance as a predictive indicator of TB treatment outcomes.

**Figure.**
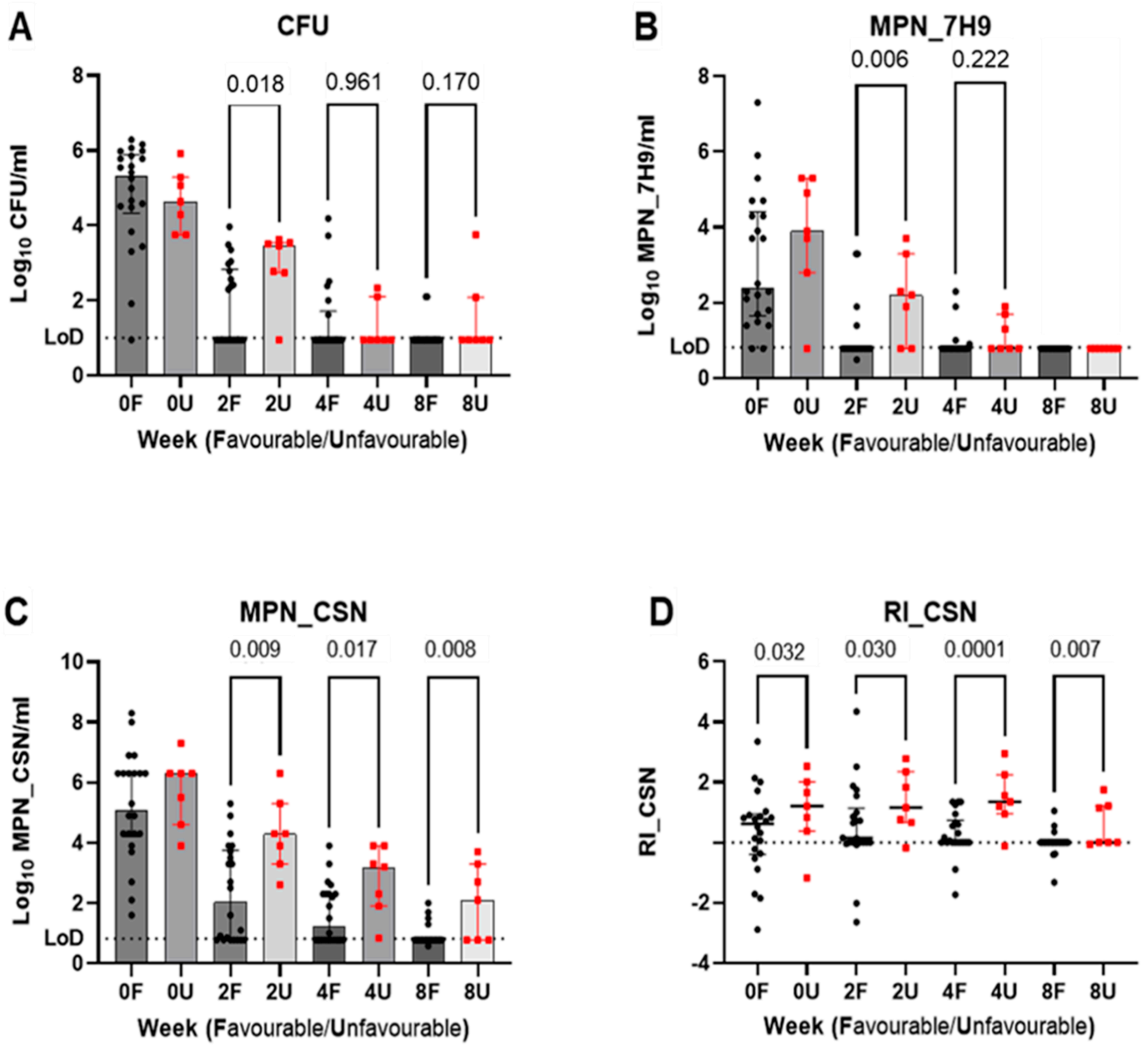
Growth and resuscitation index (RI) assay results from sputum samples taken from patients with favourable (n=22) and unfavourable (n=7) treatment outcomes. Counts from the three assay methods are shown in panels **A–C**. Note the significant proportion MPN_7H9 counts below the limit of detection (LoD) from week 2 onwards. In panel **D**, DCMtb levels are reflected in the calculated resuscitation index (RI) - Log_10_MPN_CSN - Log_10_CFU, the dotted line reflects an RI of zero; Median and interquartile ranges displayed for favourable (black symbols) and unfavourable (red symbols) outcomes. P values were determined by Mann-Whitney U test. DCMtb = differentially culturable *Mycobacterium tuberculosis*.

The TB Alliance Mark-TB Biobank (previously known as the CTB2 repository) has sputum samples from patients with fully documented treatment regimens and outcomes. In discussion with the biobank board, we first determined that DCMtb could be quantified in such samples, then agreed on the work reported here, which comprises a retrospective case-control study comparing the levels of DCMtb in serial (0, 2, 4 and 8 week) sputum samples taken from 40 sputum-positive TB patients treated with standard 2HRZE/4HR. Prior sample storage times were all in excess of 5 years.

We analysed sample sets from 29 patients, 22 with favourable and 7 with unfavourable treatment outcomes (samples from 11 individuals were excluded due to culture contamination, insufficient sample to support analysis, or failure to meet inclusion criteria). The unfavourable outcome group had clearly documented, bacteriologically confirmed re-occurrence of disease within 12 months of apparently successful treatment completion. Broadly comparable demographic and clinical features of the individuals with favourable and unfavourable outcomes are shown in the Table. Quantitative Mtb growth assays were performed by three different methods, colony forming units on agar (CFU) and limiting dilution most probable number (MPN) assays in liquid media with or without CSN supplementation (MPN_CSN and MPN_7H9, respectively). Following thawing, samples were decontaminated with NaOH, neutralised, and centrifuged pellets resuspended in Middlebrook 7H9 medium back to the original sputum volume then subjected to growth assays.^11^ Serial tenfold dilutions of the decontaminated sputum were made in 7H9 medium and in 7H9+CSN in triplicate as previously described with incubation for up to 12 weeks.^11^ The limits of detection for CFU and MPN assays were 6 and 9 propagating units/ml, respectively.

**Table.** Patient characteristics with favourable and unfavourable outcomes.

Outcome:	Favourable (n=22)	Unfavourable (n=7)
Age, median (IQR)	37.5 (31.0–35.5)	29 (27–48)
Female % (n)	23% (5)	43% (3)
Weight in kg, median (IQR)	56.2 (50.6–65.75)	51 (45.3–61.9)
BMI, median (IQR)	19 (17.66–22.35)	18.51 (18.15–23.12)
Cavitation % (n)	95% (21)^A^	100% (7)
Enrolment culture +ve (n)	100% (21)^A^	100% (7)
**Sputum smear grade at enrolment, % (n)** ^B^		
+ or scanty	32% (7)	29% (2)
++	32% (7)	57% (4)
+++	23% (5)	14% (1)

A No data available for 1 cavitation/culture;

B no data available for 3 smear results in the favourable group.

The declining patterns of counts obtained by the three growth assays are shown in Figure panels A–C. While higher MPN counts were consistently present in samples from the unfavourable group, this was most apparent with CSN addition and the RIs (Figure panel D) show a clear excess of DCMtb. This was most significant at week 4 and a ROC analysis of this data revealed an AUC of 0.805 (95% CI, 0.571–1.000, p = 0.017). Using a threshold RI value of 0.815 determined using Youden’s index, the sensitivity, specificity, positive and negative predictive values (95% CI) for unfavourable outcomes were 86% (49-99), 77% (57–90), 55% (28–79) and 94% (74–100) respectively with a diagnostic odds ratio of 20.4 (1.9–244; p=0.0055). ROC analyses of all other growth analyses (A–C) and time points showing significantly higher values in the samples from individuals with unfavourable outcomes gave AUCs less than 0.8 and lower discriminatory values.

Although this study benefitted from well-documented treatment outcomes, the numbers analysed were modest and fully powered studies will be required to validate the wider applicability of our observations. Other limitations include our use of stored samples, which raises the possibility that freezing may have differentially affected the bacillary populations enumerated (e.g. CFUs). Although we have previously demonstrated that freezing decontaminated sputum samples with addition of 10% glycerol does not significantly alter this balance,^11^ we cannot exclude a differential effect in the samples processed after the length of storage applied here. Finally, given the labour intensive and time-consuming assays applied, development of more amenable assay methods will be needed before assessment of DCMtb can be applied in the management of individual patients or in clinical trials.

Overall, we conclude that determining the Mtb resuscitation indices in stored sputum samples taken 4 weeks into treatment of pulmonary TB has promise as a means of detecting unsatisfactory outcomes, but fully powered studies are needed to validate this approach.
